# Supplementary material for: Investigating the Triple Code Model in numerical cognition using stereotactic electroencephalography
Source: PLoS One. 2024 Dec 3;19(12):e0313155. doi: 10.1371/journal.pone.0313155 (PMC11614211; doi:10.1371/journal.pone.0313155)
Supplement: Attachment — Submitted filename: Response to Reviewers.docx [file pone.0313155.s001.docx]

Dr. Kiyoshi Nakahara

Academic Editor

PLOS ONE

Dear Dr. Nakahara,

Thank you for the opportunity to revise our manuscript entitled "Investigating the Triple Code Model in Numerical Cognition Using Stereotactic Electroencephalography" (PONE-D-24-19042). We appreciate the comments and have carefully addressed each point raised with the necessary revision to enhance the quality and clarify our manuscript. Please see below for detailed response to each comment.

Sincerely,

Ahmed Raslan, MD

Professor and Interim Chair of Neurological Surgery | OHSU

Chief, Division of Functional Neurosurgery | OHSU Neurological Surgery

Thank you to both of the reviewers for your time. Both of your comments were very insightful and helped improve the quality of the manuscript. We have done our best to incorporate the changes you suggested and to provide more detail on the points that you commented on, especially in the few areas where it was not possible or practical to revise the manuscript to completely satisfy the critique. We hope that, based on the point-by-point responses below and the highlighted changes to the manuscript, we have revised our manuscript to meet the high standards of PLOS ONE in your expert opinions.

**Response to reviewer 1:**

1. Low figure Resolutions

**Response: We have replaced all figures with high resolution versions.**

a. Several fMRI studies (e.g., Masataka et al., 2007, Brain and Language; Gullick et al., 2012, NeuroImage; Hofstetter & Dumoulin, 2021, NeuroImage) have suggested the involvement of putamen which blurs the novelty of this study.

**Response:** **We agree with the important precedence of these studies and have added citations for them in the manuscript. We would defend the novelty of these claims based on the time-frequency resolution of electrophysiological measurements and the important differences in task design, including our inclusion of a wide array of modalities in which the numbers were presented. The addition to the manuscript in the conclusion is included below (line 385-390):**

**Previous studies have demonstrated the involvement of the putamen in visual processing of Roman numerals (Masataka, 2007), magnitude estimates of negative numbers (Gullick, 2012) and haptic numerosity (Hofstetter & Dumoulin, 2022). Our findings not only replicate the involvement of the putamen in processing of numerals but also provide detail about how the putamen responds to different modality presentations of numbers and the time-frequency characteristics of those responses.**

b. [The] introduction should better justify the need for sEEG over fMRI to understand subcortical contributions. To emphasize the advantages of sEEG, discuss the contributed latencies and frequencies in more detail

**Response: We added the following sentences in the introduction to address this point. Given recent and past findings questioning reproducibility in the field of fMRI neuroimaging, we think that the importance confirmation by another neuroimaging modality is justified. And sEEG detects fast changes in neural activity like oscillations, providing additional information to fMRI. Thank you for this comment, adding these changes makes it more clear to the reader the importance of this research. (line 83-91)**

**Comparing sEEG to functional magnetic resonance imaging (fMRI), both record from the same sub-surface brain structures, however, fMRI measures blood oxygenation whereas sEEG is a direct measure of electrical neural communication. Practically, this is important because of reproducing brain-wide association studies using fMRI has been shown to take thousands of individuals (Marek, 2022) so having corroborating evidence from other neuroimaging modalities is essential, and sEEG can detect millisecond-time scale changes in neural activity whereas fMRI samples activity on the order of every two seconds. Thus, sEEG can detect the precise order of brain area activation as well as fast neural activity such as oscillations.**

c. Discussion mentions the relations between fMRI BOLD and high-gamma, weakens the findings because new findings of high-gamma contribution can be well predicted in the literature

**Response:** **This is a very reasonable critique. While fMRI is incredibly valuable and has made the most progress to date in understanding the neural correlates of numerical cognition, fMRI is not perfect and sEEG nicely complements those imperfections with better time-resolution, including differentiating when the high-gamma activity occurred relative to the presentation of the stimulus, and high signal-to-noise allowing us to present many different stimulus modalities in a relatively short period of time. Thus, we posit that, although the information presented in this study is not as foundational as early fMRI studies, the novel details from this study are still important and may be essential pieces of information for piecing together the more nuanced aspects of numerical cognition.**

3. Difficult to understand the consistency with TCM without discussing how these regions align with or differ from TCM and prior studies

**Response:** **The aim of this study was to re-examine the basis of the triple code model as originally postulated using sEEG. Thus our focus was on the very basic replication of inferior temporal gyrus as visual number form area, superior temporal gyrus as verbal number language processing and intraparietal sulcus as non-symbolic number processing area and to explore how structures outside these triple code areas are involved. Because we did not specifically set out to study the involvement of the putamen or other subcortical structures, rather to see which structures were found to be involved based on the significance of statistical quantification of the classifications, we decided not to revise the introduction to include these structures so as to attempt to present our scientific process as it actually occurred. We did clarify this point in the end of the introduction so that this lack of clarity will not cause issues for readers. We also added comparisons to the original triple code model as well as later work extending this model in the discussion.**

**This aligns with the frontoparietal network of number cognition posited by Dehaene et al. and the hypothesized role of the parietal cortex put forth by the TCM (Dehaene & Cohen, 1995; Dehaene, 2003) with the notable difference that the right parietal sEEG channels had lower classification accuracy, questioning the whether the right-hemisphere dominance found in previous studies should be re-examined.**

4. Current method may not be optimal for identifying critical brain regions from different numerical representation forms, and classification across all stimulus forms would better highlight the differences in neural processing

**Response: This is an excellent point, but unfortunately, the resolution of brain activity for current sEEG recording technology is apparently not up to this level of specificity. We analyzed but did not present decoding the numerical quantity and <= 3 vs. > 3 based on previous work by Dehaene showing behavioral differences in the recognition of three or fewer compared to more than three but no channels decoded these features with significant accuracy. This is likely due to the relatively small number of neurons that are tuned to specific numerical quantity which are not numerous or synchronous enough to be detected by sEEG, compared to the greater number of neurons that are tuned to number, possibly with an ideal response to a particular number, such that for numeric stimuli there is greater synchronous activation compared to during the inter-trial interval. Our group is involved in developing higher-resolution recordings for this purpose and so we have added this future direction to the discussion as well as adding transparency of analyses with null results.**

5. Comment 5:
   a. A. SVM classification with PCA has not well described leading to confusion about the output of analyses

**Response: The SVM classification using PCA components is quite complicated, and we revised the caption for Figure 2 to ameliorate this issue. In brief, this technique was used because the simplicity of the SVM makes it interpretable but it is not able to converge due to the size of the spectrogram data relative to the number of trials. Dimensionality reduction in the form of PCA, allows the SVM to converge efficiently and yields stable, generalizable results. Thank you for bringing this lack of a high-level overview of this method to our attention, we have also a similar summary to the methods of the text for the reader.**

b. The explanation of null distribution

**Response: The null distribution is a binomial with probability 0.5 corresponding to the 0.5 probability the stimulus-inter-trial interval classification being correct due to chance given the number of trials that the particular number modality was shown. This explanation was added in the caption of Fig 3 and in the Methods section. This distribution was found to be nearly identical to dividing up the inter-trial interval into two non-overlapping segments and classifying whether the data came from the first or second segment. In an earlier version of the results, the null classification distribution was used, but in the figures in the current version of the manuscript, the binomial distribution was used. This has been corrected in the Methods section of the text.**

6. Clarify whether classification accuracies were cross validated. Recommendation: applying cross validation by repeating the computation multiple times to ensure robustness

**Response:** **This is an excellent point to ensure robust statistical analysis. In this case, the results of the cross-validation analysis were very similar as shown below. Consequently, we added a sentence in the methods endorsing cross-validation and explaining that the results were similar in this case.**

**A 70/30% training-test split was used. This yielded results that were found to be similar to using six-fold cross-validation.**

**Minor comments:**

1. Comment 1:
   a. Interpretation of left parietal cortex in processing numerical dots as aligning with “the hypothesized role of the left parietal [cortex] put forth by the TCM." Did Dehaene specifically limit this role to the left side?

**Response: Dehaene suggested that both hemispheres are involved and, in fact, that the right side may even be dominant (excerpt below). Since we found greater involvement in the left hemisphere, we added an explanation of this important difference in the text. Thank you for catching this!**

b. "rgcortex" appears to be a typo.

**Response: “rgcortex” was corrected to “cortex”.**

2. Figure 9 lacks explanation of the color code used

**Response: The cluster permutation test between the inter-trial interval and stimulus period is difficult to interpret because it has both a sign (increased or decreased during the stimulus) and a proportion of channels for which that particular time-frequency is significant. To show both pieces of information, the first row what proportion of channels are significant at that time-frequency ranging from 0 to 1, and the second row shows which proportion are positive ranging from 0 to 1. This way, if there are time-frequencies that increase in some locations and decrease in others, they will still be shown in yellow in the first row, they will just be an intermediate green color in the second row. This explanation has been added to the caption and colorbars have been added to the figure.**

3. Line 52: "Triple Code Model" (TCM) seems to be incorrectly abbreviated as "TMC."

**Response: “TMC” was corrected to “TCM”.**

4. There are inconsistencies in spelling "sEEG contacts" and "sEEG channels"

**Response: “sEEG contacts” were changed to “sEEG channels”.**

**Reviewer 2:**

1. The literature review could help tell more about the sEEG and why it might be better than other mediums such as TMS and ECoG.

   **Response: We added details to the introduction about the specific brain areas that sEEG records from that ECoG does not, and we added details about the specificity of recording and stimulation of sEEG relative to scalp EEG and TMS in the third paragraph of the Introduction.**
2. Providing more information about the methodology, including the study design and parameters, would enhance the clarity and replicability of the research

**Response: We added details about the task such as the exact proportion of catch trials and the duration of the stimulus period. Furthermore, we added a link to the GitHub repository with the task code so that readers can inspect all the details of the task and try it themselves.**

3. I would recommend showing some power statistics for the results, which could provide more credibility to the findings and ensure the study's conclusions are supported by robust data analysis.

**Response: This is a great point. We added the following text to the Methods section to quantify our experimental power.**

***With 40 trials per number modality, we were powered at 79.97% to detect a large effect size of 0.4 between the stimulus and inter-trial interval conditions.***
